# Supplementary material for: Archaeal DNA-import apparatus is homologous to bacterial conjugation machinery
Source: Nat Commun. 2023 Feb 7;14:666. doi: 10.1038/s41467-023-36349-8 (PMC9905601; doi:10.1038/s41467-023-36349-8)
Supplement: Supplementary file 1 — Supplementary Information [file 41467_2023_36349_MOESM1_ESM.pdf]

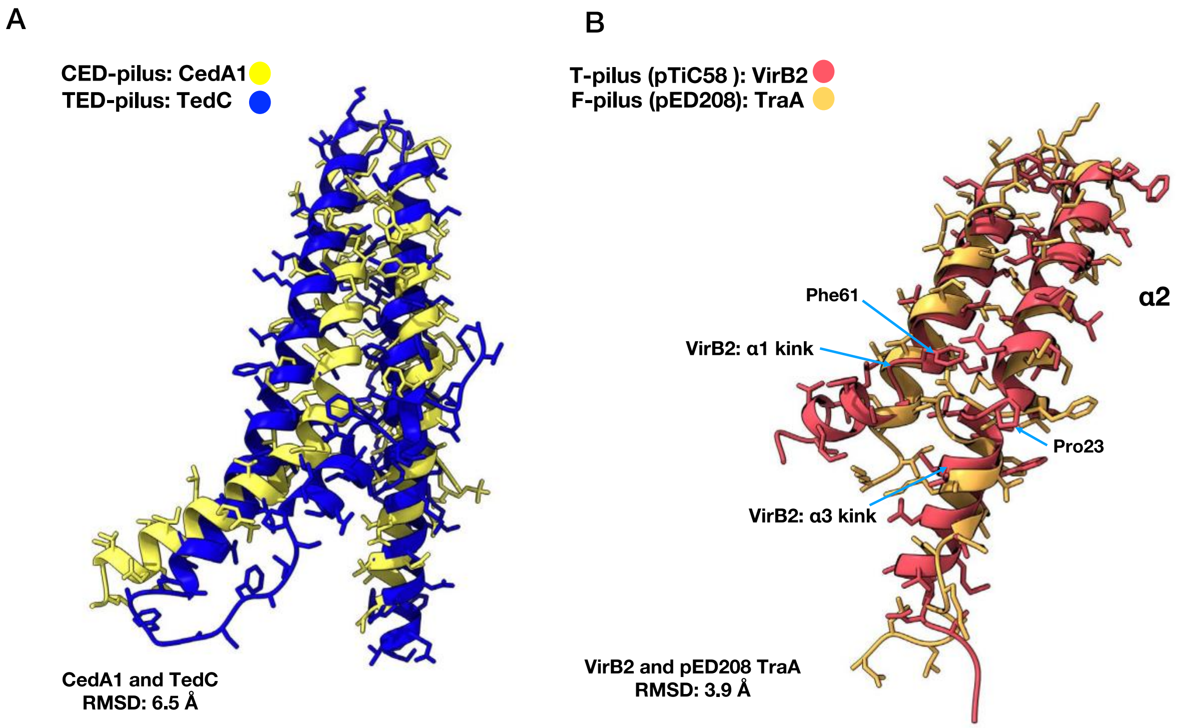

Supplementary Fig. 1. (A) An alignment of the CedA1 (yellow) with the TedC (blue) subunits shows an RMSD of 6.5 Å between the two subunits. A distinct kink in helix α2 of CedA1, while the kink in helix α2 of TedC is present but less prominent. **(B)** Alignment of the VirB2 subunit (red) with the TraA subunits of pED208 (orange). There is a 3.9 Å RMSD between VirB2 and pED208 across 58 atom pairs. There are two distinct kinks in VirB2 not present in pED208. The kink in VirB2 helix α1 occurs at phenylalanine 61 and the kink in α3 occurs at proline 23.

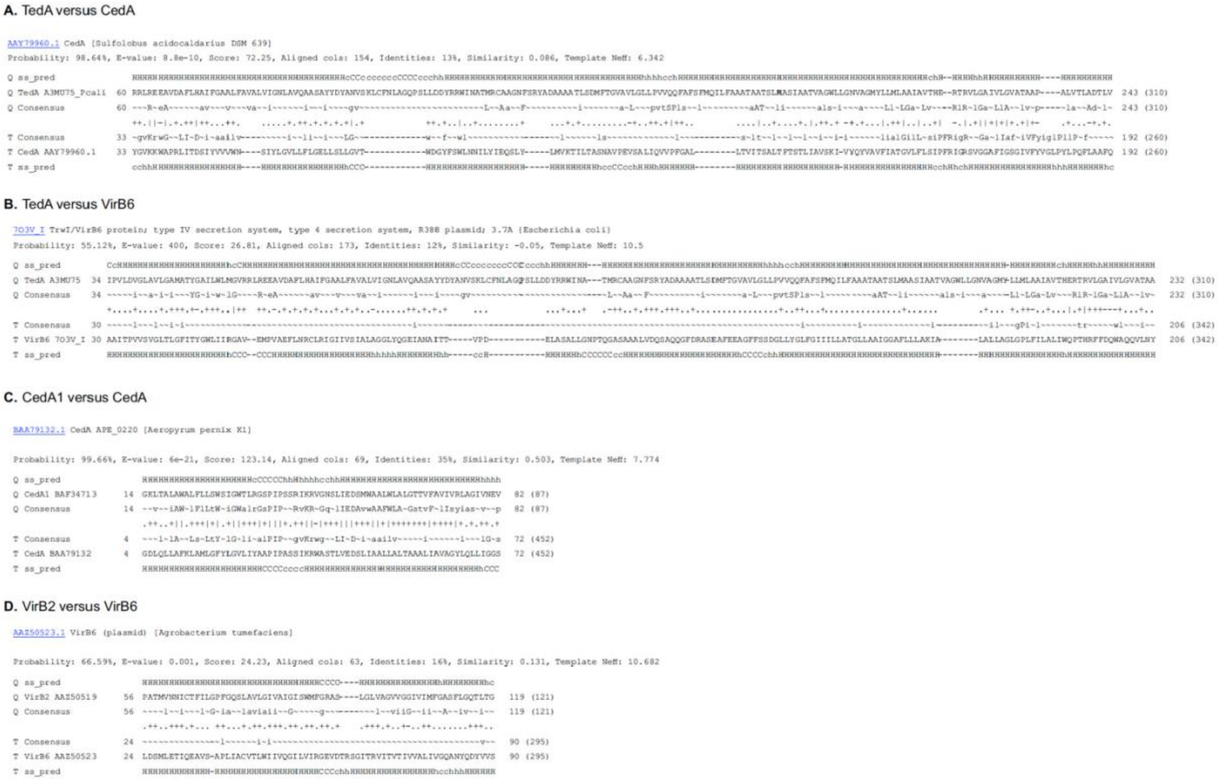

Supplementary Fig. 2. Results of the profile-profile comparisons using HHsearch for different components of the conjugative machineries of bacteria and archaea. **(A)** Comparison of the archaeal membrane pore proteins TedA and CedA. **(B)** Comparison of the archaeal protein TedA and bacterial protein VirB6. **(C)** Comparison of CedA and CedA1. **(D)** Comparison of bacterial conjugation proteins VirB2 and VirB6. H(h), α-helix; E(e), β-strand; C(c), coil.

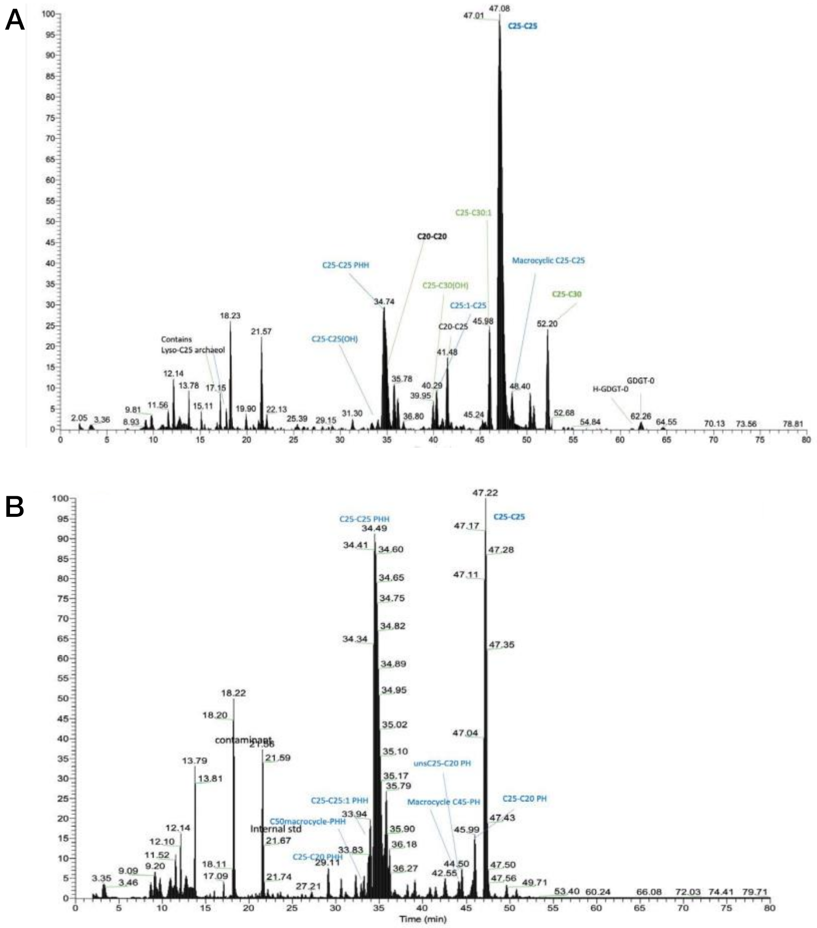

Supplementary Fig. 3. Mass spectrometry of the lipids extracted from *A. pernix* cell culture. **(A)** A chromatogram of the core lipids found in the sample showing C25-C25 diether lipids as the most abundant species. **(B)** A chromatogram of the most abundant lipid headgroups reveals phospho-dihexose, i.e., a dihexose sugar connected to the glycerol by a phosphate group, as the most prevalent lipid headgroup and phospho-hexose, one sugar connected via a phosphate-group, as the second most prevalent.

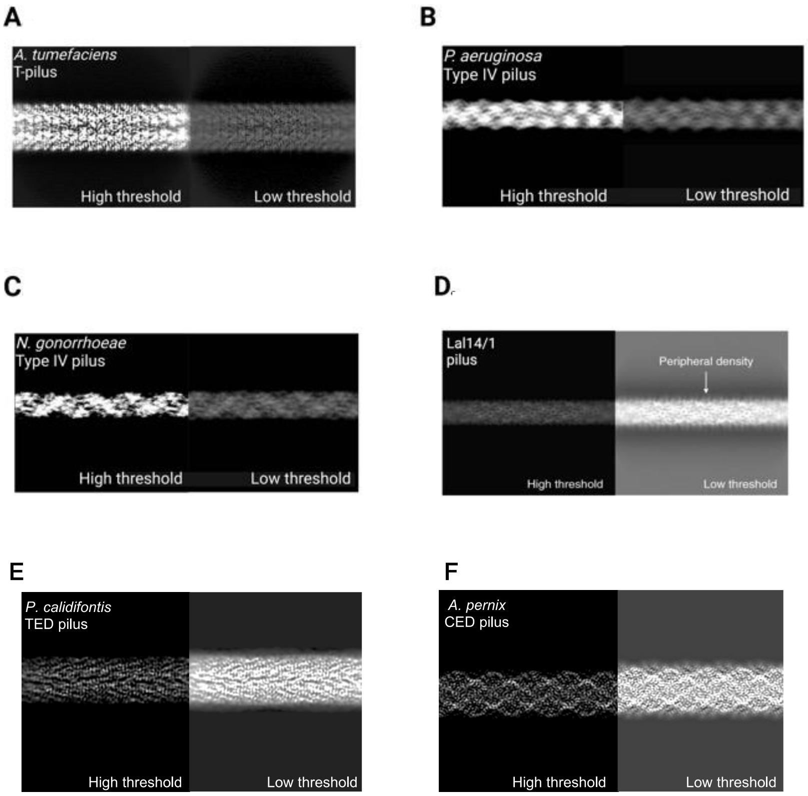

Supplementary Fig. 4. Projections of the 3D reconstructions at different thresholds reveal if pili are extensively glycosylated. T4P pili of *Neisseria gonorrhoeae* **(A,** EMDB deposition EMD-8739, [https://www.emdataresource.org/EMD-8739]**)** and *Pseudomonas aeruginosa* **(B,** EMDB deposition EMD-8740, [https://www.emdataresource.org/EMD-8740]**)** are shown as negative controls, whereas the highly glycosylated type IV pilus of *Saccharolobus islandicus* LAL14/1 **(C,** EMDB deposition EMD-0397, [https://www.emdataresource.org/EMD-0397]**)** shows peripheral density at low threshold, and serves as a positive control. Both the Ted pilus of *P. calidifontis* **(D)** and the Ced pilus of *A. pernix* **(E)** show some surrounding density, which could represent glycosylation. In contrast, the T-pilus of *A. tumefaciens* **(F)** shows no obvious surrounding density at low threshold and behaves like controls A and B.

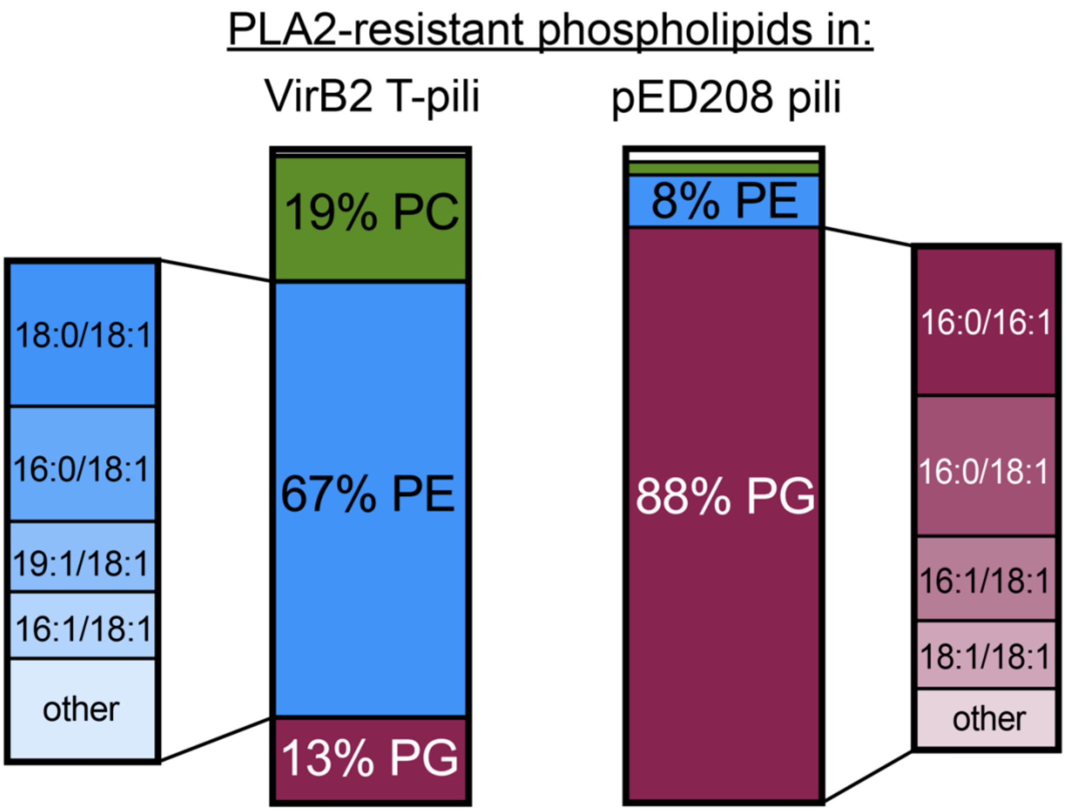

Supplementary Fig. 5. PLA2 was applied to purified pili from VirB2 and pED208 to digest any unprotected phospholipids. The remaining lipids were extracted, then identified by mass spectrometry and quantified using internal standards. The major phospholipid classes in the pili samples are shown by headgroup class, and the acyl chain compositions are shown for the major headgroup classes (PE for VirB2, PG for pED208). Unlabeled bars represent minor species.

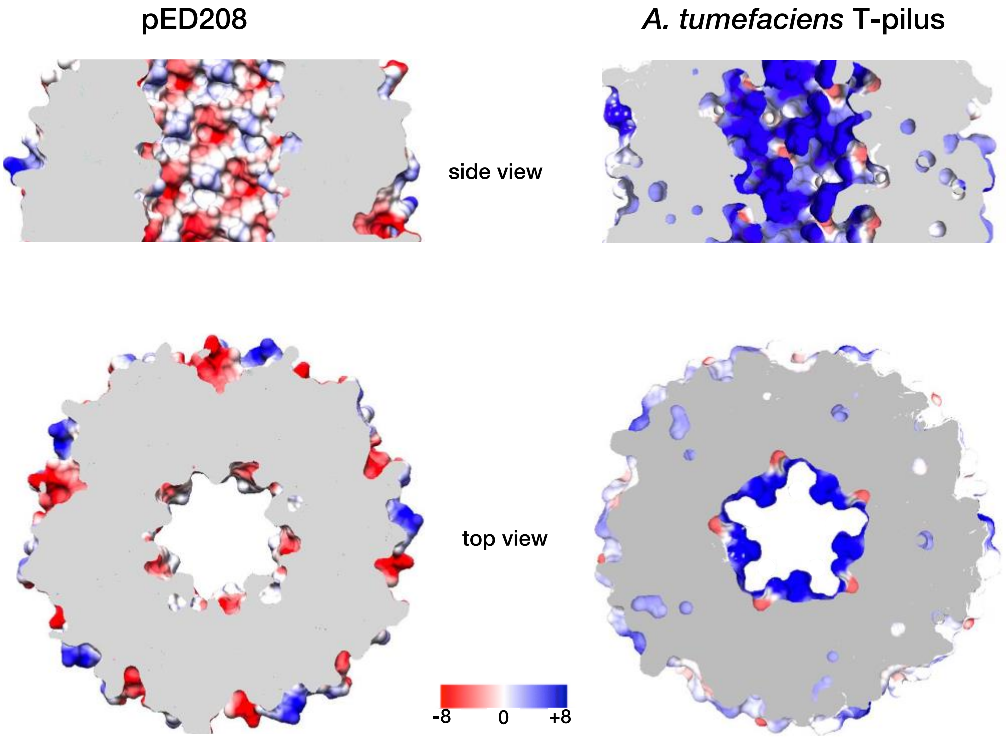

Supplementary Fig. 6. Electrostatic potential surface for the lumen of the pED208 (left) and T-pilus (right). The side and top view of the pED208 pilus shows a more electronegative lumen compared to the T-pilus. The top view of the T-pilus shows an alternating positive to negative charge arising from the arginine and the lipid, respectively. All electrostatic potentials and surfaces were calculated using UCSF Chimera. The units for the potential are kT/e.

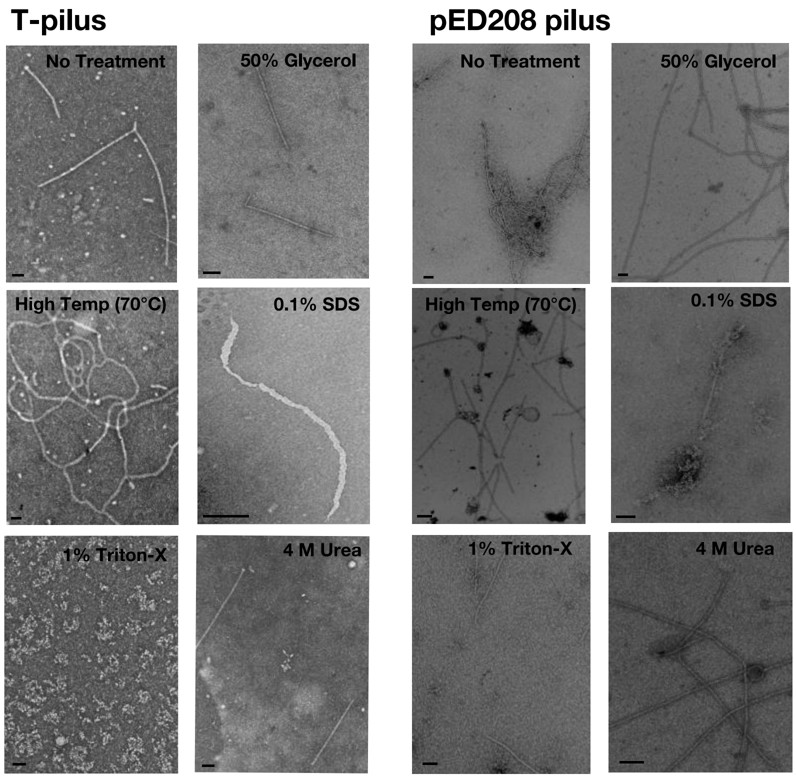

Supplementary Fig. 7. Negative stain electron micrographs showing the effects on pilus structure of different harsh environmental conditions for *A. tumefaciens* T-pilus (left) and *E. coli* pED208 (right), scale bars 50 nm. The morphology of the T-pilus under no treatment, glycerol (50%) and 4M urea appears fully intact. The high-temperature (70°C) and SDS (0.1%) partially degrades the pilus and results in flexuous filaments. Triton-X (1%) fully depolymerizes the filament and creates aggregated clusters. The pED208 pilus morphology is mostly in a flexuous state with partial bundling at the ends of the pilus under SDS (0.1%). Each condition/treatment was replicated three times for both the T-pilus and pED208. Over 15 grid squares were chosen at random and examined for each sample to ensure a broad representation of pilus morphology across the entire electron microscopy grid. See Table S2 for more details.

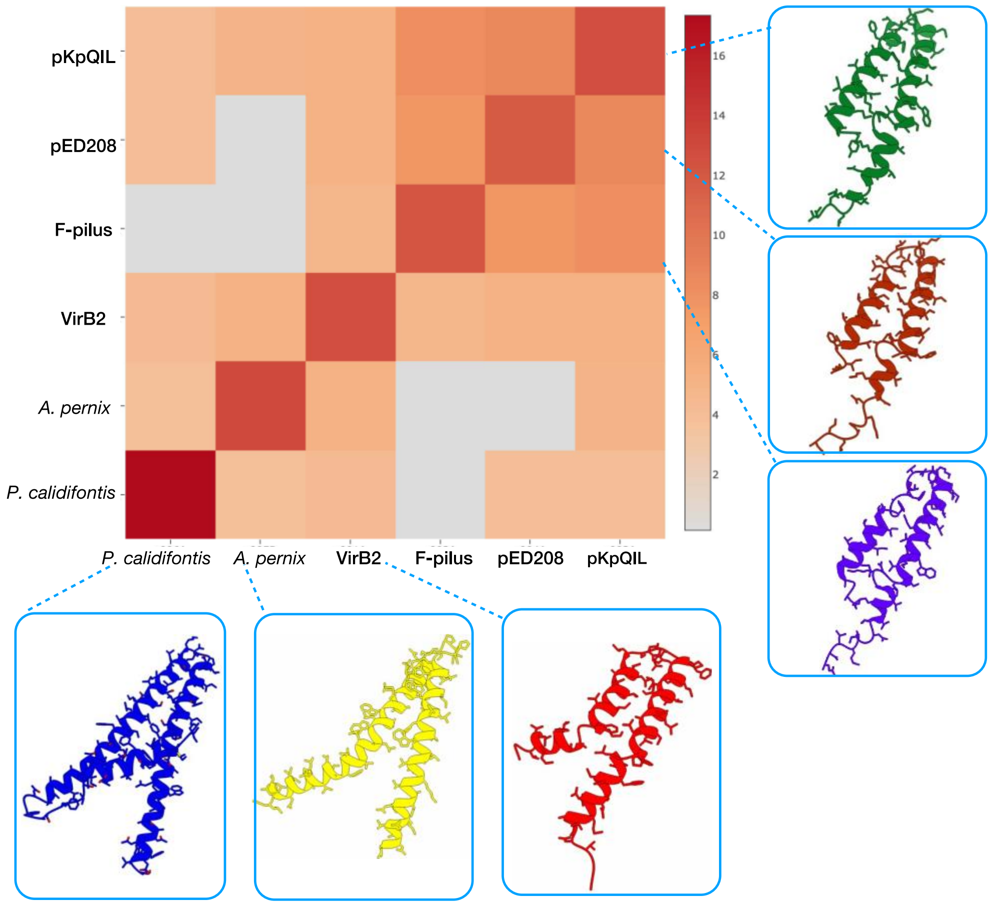

Supplementary Fig. 8. Heat-map of global structural similarity between pilins from all known structures of prokaryotic conjugation pili: *P. calidifontis* (blue), *A. pernix* (yellow), *A. tumefaciens* (red), *F-pilus* (purple), *pED208* (light brown) and *pKpQIL* (green). The color scale corresponds to the Dali Z-score values. The dark red indicates increased similarity, the lighter red indicates some similarity and grey is no to low structure similarity. Each pilin consists of two or three hydrophobic α-helices with kinks appearing in both of the archaeal pili and the *A. tumefaciens* T-pilin. All share a common helix-turn-helix architecture and have obvious structural homology.

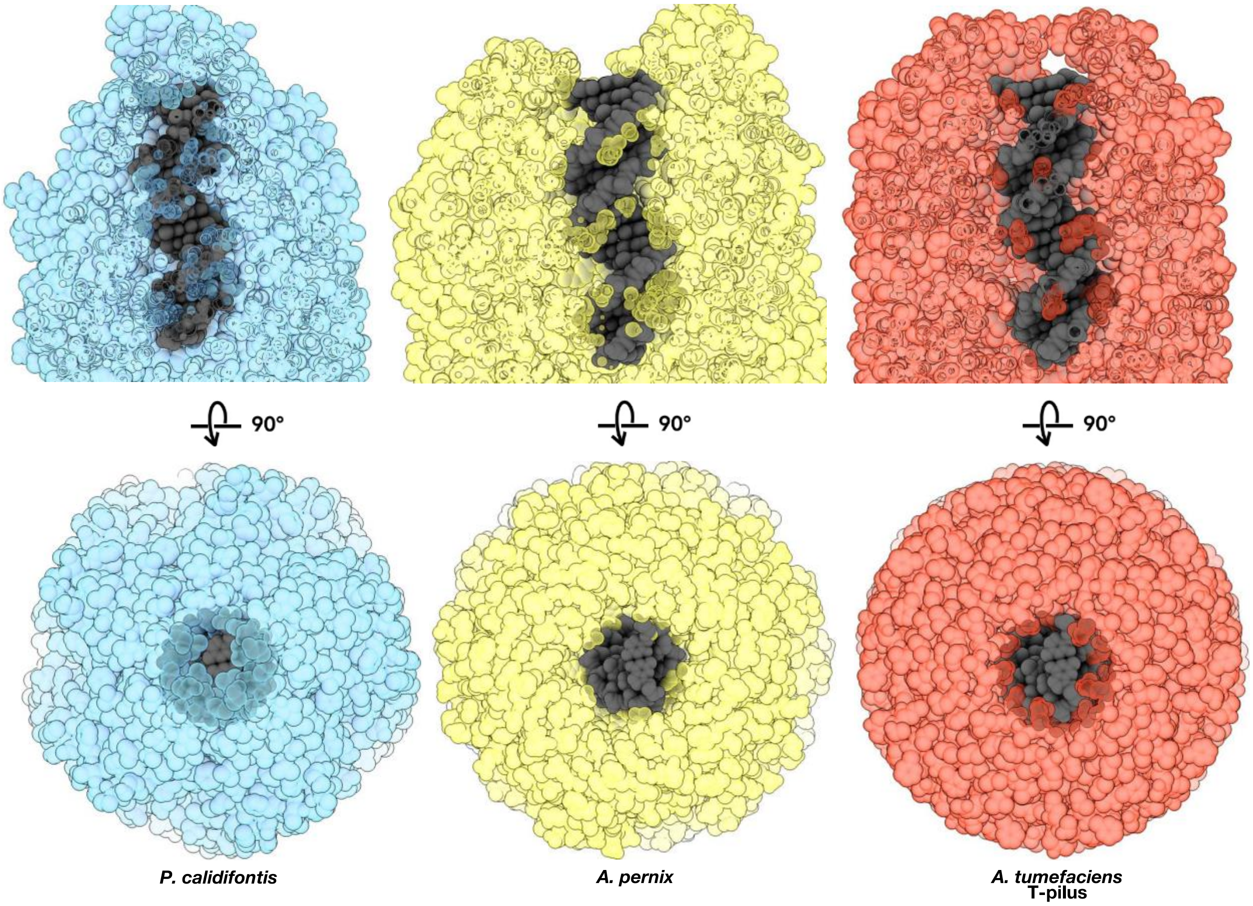

Supplementary Fig. 9. Conjugative pili cannot transport dsDNA. Side views (top) and top views (bottom) of the atomic models of *P. calidifontis* (blue), *A. pernix* (yellow), and *A. tumefaciens* (red) with a model for B-form dsDNA placed within the lumen. The narrow lumen of all the prokaryotic conjugative pili would result in extensive clashes with dsDNA suggesting that only ssDNA could be accommodated within the lumen. The atomic model of *P. calidifontis* (blue) does not include a lipid model with phospho-dihexose headgroups. The addition of these sugar headgroups would decrease the pore size and result in even further clashes with dsDNA.

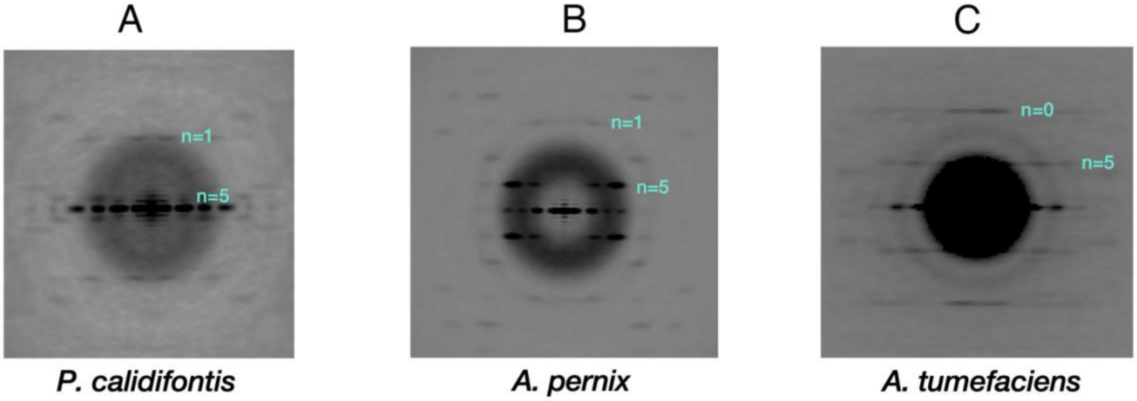

Supplementary Fig. 10. Averaged power spectra of the *P. calidifontis, A. pernix* and *A. tumefaciens* pili. (A.) Averaged power spectrum generated for *P. calidifontis* from 54,000 segments. Two layer lines are indexed with their Bessel orders: the n=1 and n=5. **(B.)** Averaged power spectrum generated for *A. pernix* from 44,000 segments. Two layer lines are indexed with their Bessel orders: the n=1 and n=5. **(C.)** Averaged power spectrum generated for *A. tumefaciens* from 49,000 segments. Two layer lines are indexed with their Bessel orders: the n=0 and n=5. To increase the dynamic range, the log of the intensities are shown for *P.calidifontis* and *A. tumefaciens*.

Table S1. Cryo-EM and refinement statistics.

Parameter	*P. calidifontis* TedC	*A. pernix* CedA1	*A. tumefaciens* VirB2
**Data collection and processing**			
Voltage (kV)	300	300	300
Electron exposure ($e^{-}Å^{-2}$)	50	50	50
Pixel size (Å)	1.08	1.08	1.08
Segments (n)	71,981	44,262	49,308
**Helical symmetry**			
Point group	C1	C1	C5
Helical rise (Å)	5.00	3.61	13.68
Helical twist (°)	74.21	76.5	32.45
**Map resolution (Å)**			
Software (final reconstruction)	cryoSPARC	cryoSPARC	cryoSPARC
Model:map FSC (0.38)	4.2	3.5	3.7
Map:map FSC (0.143)	4.0	3.3	3.5
**Refinement and Model validation**			
Bond lengths rmsd (Å)	0.004	0.007	0.003
Bond angles rmsd (°)	0.796	0.612	0.632
Clashscore	16.28	3.45	4.3
Ramachandran Favored (%)	97.27	98.78	100
Ramachandran Outlier (%)	0	0	0
MolProbity score	1.85	1.14	1.66
**Deposition ID**			
PDB (model)	8DFT	8DFU	8EXH
EMDB (map)	EMD-27413	EMD-27414	EMD-28657

Table S2. Environmental effects on pilus structure.

Environmental effects on T-pilus and pED208 structure

Agent or condition	Temperature (°C)	Morphology of T-pilus filament[1]	Morphology of pED208 pilus filament[1]
No Treatment	23	Rigid/Fully intact	Flexuous/Fully intact
Glycerol (50%)	23	Rigid/Fully intact	Flexuous/Fully intact
High temperature	70	Flexuous/Partially degraded	Flexuous/Fully intact
SDS (0.1%)	23	Flexuous/Partially degraded	Bundling at the ends of the pilus /Fully intact
Triton X-100 (1%)	23	Aggregated clusters/Fully depolymerized	Flexuous/Fully intact
Urea (4 M)	23	Rigid/Fully intact	Flexuous/Fully intact

[1]For each preparation the sample was negatively stained with 2% uranyl acetate and examined by transmission electron microscopy

Table S3. Standard Lipid Mixtures

Cer 35:1;2, (D18:1;2, 17:0;0)
Chol D6
DAG 34:0;0 (17:0;0, 17:0;0)
DiHexCer 30:1;2 (D18:1;2.12:0;0)
HexCer 30:1;2 (D18:1;2.12:0;0)
LPA 17:0;0 (17:0;0)
LPC 12:0;0 (12:0;0)
LPE 17:1;0 (17:1;0)
LPI 17:1;0 (17:1;0)
LPS 17:1;0 (17:1;0)
PA 34:0;0 (17:0;0, 17:0;0)
PC 34:0;0 (17:0;0, 17:0;0)
PE 34:0;0 (17:0;0, 17:0;0)
PG 34:0;0 (17:0;0, 17:0;0)
PI 32:0;0 (16:0;0, 16:0;0)
PS 34:0;0 (17:0;0, 17:0;0)
SE 20:0;0 (20:0;0)
SM 30:1;2 (18:1;2, 12:0;0)
TAG 51:0;0 (17:0;0, 17:0;0, 17:0;0)
