# Supplementary material for: Archaeal DNA-import apparatus is homologous to bacterial conjugation machinery
Source: Nat Commun. 2023 Feb 7;14:666. doi: 10.1038/s41467-023-36349-8 (PMC9905601; doi:10.1038/s41467-023-36349-8)
Supplement: Supplementary file 3 — Reporting Summary [file 41467_2023_36349_MOESM3_ESM.pdf]

nature portfolio

Corresponding author(s): Edward H. Egelman, Mart Krupovic

Last updated by author(s): 20 January 2023

Reporting Summary

Nature Portfolio wishes to improve the reproducibility of the work that we publish. This form provides structure for consistency and transparency in reporting. For further information on Nature Portfolio policies, see our Editorial Policies and the Editorial Policy Checklist.

Statistics

For all statistical analyses, confirm that the following items are present in the figure legend, table legend, main text, or Methods section.

n/a	Confirmed	
☒	☐	The exact sample size (*n*) for each experimental group/condition, given as a discrete number and unit of measurement
☒	☐	A statement on whether measurements were taken from distinct samples or whether the same sample was measured repeatedly
☒	☐	The statistical test(s) used AND whether they are one- or two-sided<br>*Only common tests should be described solely by name; describe more complex techniques in the Methods section.*
☒	☐	A description of all covariates tested
☒	☐	A description of any assumptions or corrections, such as tests of normality and adjustment for multiple comparisons
☒	☐	A full description of the statistical parameters including central tendency (e.g. means) or other basic estimates (e.g. regression coefficient) AND variation (e.g. standard deviation) or associated estimates of uncertainty (e.g. confidence intervals)
☒	☐	For null hypothesis testing, the test statistic (e.g. *F*, *t*, *r*) with confidence intervals, effect sizes, degrees of freedom and *P* value noted<br>*Give P values as exact values whenever suitable.*
☒	☐	For Bayesian analysis, information on the choice of priors and Markov chain Monte Carlo settings
☒	☐	For hierarchical and complex designs, identification of the appropriate level for tests and full reporting of outcomes
☒	☐	Estimates of effect sizes (e.g. Cohen's *d*, Pearson's *r*), indicating how they were calculated

Our web collection on statistics for biologists contains articles on many of the points above.

Software and code

Policy information about availability of computer code

Data collection	EPU (ThermoFisher) for EM data collection, version 2.5.0.4799REL
Data analysis	CryoSPARC v3.3, Molprobity

For manuscripts utilizing custom algorithms or software that are central to the research but not yet described in published literature, software must be made available to editors and reviewers. We strongly encourage code deposition in a community repository (e.g. GitHub). See the Nature Portfolio guidelines for submitting code & software for further information.

Data

Policy information about availability of data

All manuscripts must include a data availability statement. This statement should provide the following information, where applicable:

- Accession codes, unique identifiers, or web links for publicly available datasets
- A description of any restrictions on data availability
- For clinical datasets or third party data, please ensure that the statement adheres to our policy

The atomic model for the P. calidifontis pilus was deposited in the Protein Data Bank with accession code 8DFT [https://www.rcsb.org/structure/8DFT], and the corresponding map was deposited in the Electron Microscopy Data Bank with accession code EMD-27413 [https://www.emdataresource.org/EMD-27413]. The atomic model for the A. pernix pilus was deposited in the Protein Data Bank with accession code 8DFU [https://www.rcsb.org/structure/8DFU], and the corresponding map was deposited in the Electron Microscopy Data Bank with accession code EMD-27414 [https://www.emdataresource.org/EMD-27414]. The atomic model for the A. tumefaciens pilus was deposited in the Protein Data Bank with accession code 8EXH [https://www.rcsb.org/structure/8EXH], and the

corresponding map was deposited in the Electron Microscopy Data Bank with accession code EMD-28657 [https://www.emdataresource.org/EMD-28657].

Human research participants

Policy information about studies involving human research participants and Sex and Gender in Research.

Reporting on sex and gender	N/A
Population characteristics	N/A
Recruitment	N/A
Ethics oversight	N/A

Note that full information on the approval of the study protocol must also be provided in the manuscript.

Field-specific reporting

Please select the one below that is the best fit for your research. If you are not sure, read the appropriate sections before making your selection.

☒ Life sciences ☐ Behavioural & social sciences ☐ Ecological, evolutionary & environmental sciences

For a reference copy of the document with all sections, see nature.com/documents/nr-reporting-summary-flat.pdf

Life sciences study design

All studies must disclose on these points even when the disclosure is negative.

Sample size	71,981, 44,262, and 49,308 filament segments were used for P. calidifontis, A. pernix, and A. tumefaciens, respectively.
Data exclusions	Micrographs were excluded that yielded a poor fit in CTF determination.
Replication	Each 3D reconstruction involved two independent data sets, which can be viewed as replication.
Randomization	The images were randomized
Blinding	This is impossible

Reporting for specific materials, systems and methods

We require information from authors about some types of materials, experimental systems and methods used in many studies. Here, indicate whether each material, system or method listed is relevant to your study. If you are not sure if a list item applies to your research, read the appropriate section before selecting a response.

Materials & experimental systems

n/a	Involved in the study	
☒	☐	Antibodies
☒	☐	Eukaryotic cell lines
☒	☐	Palaeontology and archaeology
☒	☐	Animals and other organisms
☒	☐	Clinical data
☒	☐	Dual use research of concern

Methods

n/a	Involved in the study	
☒	☐	ChIP-seq
☒	☐	Flow cytometry
☒	☐	MRI-based neuroimaging
